# Imaging Mass Cytometric Analysis of Postmortem Tissues Reveals Dysregulated Immune Cell and Cytokine Responses in Multiple Organs of COVID-19 Patients

**DOI:** 10.3389/fmicb.2020.600989

**Published:** 2020-12-23

**Authors:** Chong Wang, Jiqian Xu, Shaoyuan Wang, Shangwen Pan, Jiancheng Zhang, Yang Han, Muhan Huang, Di Wu, Qingyu Yang, Xiaobo Yang, Yang Yang, Ting Shu, Xiaojing Zou, Ruiting Li, Yufeng Luo, Runqing Yao, Yaxin Wang, Yang Qiu, Yu Wang, Ding-Yu Zhang, Qun Yao, Yongpan Yan, Xi Zhou, You Shang

**Affiliations:** ^1^Center for Precision Translational Medicine of Wuhan Institute of Virology & Guangzhou Women and Children's Medical Center, Guangzhou Women and Children's Medical Center, Guangzhou, China; ^2^Joint Laboratory of Infectious Diseases and Health, Wuhan Institute of Virology & Wuhan Jinyintan Hospital, Chinese Academy of Sciences, Wuhan, China; ^3^Department of Critical Care Medicine, Tongji Medical College, Union Hospital, Huazhong University of Science and Technology, Wuhan, China; ^4^Gencode Diagnostics Inc., Beijing, China; ^5^State Key Laboratory of Virology, Center for Biosafety Mega-Science, Chinese Academy of Sciences, Wuhan Institute of Virology, Wuhan, China; ^6^Center for Translational Medicine, Wuhan Jinyintan Hospital, Wuhan, China; ^7^Life Science Institute, Jinzhou Medical University, Jinzhou, China; ^8^Tongji Medical College, Institute of Anesthesiology and Critical Care Medicine, Union Hospital, Huazhong University of Science and Technology, Wuhan, China

**Keywords:** imaging mass cytometry (IMC), COVID-19, organ specific response, immune dysregulation, inflammatory response

## Abstract

SARS-coronavirus-2–induced immune dysregulation and inflammatory responses are involved in the pathogenesis of coronavirus disease-2019 (COVID-19). However, very little is known about immune cell and cytokine alterations in specific organs of COVID-19 patients. Here, we evaluated immune cells and cytokines in postmortem tissues, i.e., lungs, intestine, liver, kidneys, and spleen of three patients with COVID-19. Imaging mass cytometry revealed monocyte, macrophage, and dendritic cell (DC) infiltration in the lung, intestine, kidney, and liver tissues. Moreover, in patients with COVID-19, natural killer T cells infiltrated the liver, lungs, and intestine, whereas B cells infiltrated the kidneys, lungs, and intestine. CD11b^+^ macrophages and CD11c^+^ DCs also infiltrated the lungs and intestine, a phenomenon that was accompanied by overproduction of the immunosuppressive cytokine interleukin (IL)-10. However, CD11b^+^ macrophages and CD11c^+^ DCs in the lungs or intestine of COVID-19 patients did not express human leukocyte antigen DR isotype. In contrast, tumor necrosis factor (TNF)-α expression was higher in the lungs, intestine, liver, and kidneys, but not in the spleen, of all COVID-19 patients (compared to levels in controls). Collectively, these findings suggested that IL-10 and TNF-α as immunosuppressive and pro-inflammatory agents, respectively,—might be prognostic and could serve as therapeutic targets for COVID-19.

Coronavirus disease-2019 (COVID-19) caused by severe acute respiratory syndrome coronavirus 2 (SARS-CoV-2) has become a global pandemic and the worst public health crisis at present. COVID-19 has been reported to be associated with diverse co-morbidities, such as pneumonia, respiratory failure, acute respiratory distress syndrome, and sepsis, that are usually associated with pathophysiological changes, including alveolar macrophage activation, lymphopenia, and thrombosis (Chen et al., 2020; Fu et al., 2020; Guan et al., 2020; Huang et al., 2020; Jose and Manuel, 2020; Moore and June, 2020; Wang et al., 2020; Yang et al., 2020). Additionally, in addition to the lower respiratory tract and lungs, SARS-CoV-2 targets many other organs, including the liver, heart, intestine, kidneys, central nervous system, and muscles, thereby resulting in multiorgan injury, which is frequently associated with severe or even fatal outcomes (De Felice et al., 2020; Varga et al., 2020; Zhang C. et al., 2020). Immune dysregulation and inflammation are therefore involved in the pathogenesis of COVID-19 (Giamarellos-Bourboulis et al., 2020; Moore and June, 2020). However, most of the information regarding the dysregulation of immune cells and cytokines has been obtained from studies using blood samples from patients. Therefore, it is essential to profile COVID-19-associated immune cell and pro-inflammatory cytokine responses in specific organs and tissues to improve our understanding of the pathogenesis of this disease.

Tissue samples from COVID-19 patients including the lungs, intestine, spleen, liver, and kidneys, were obtained from three deceased COVID-19 patients. Paracancerous tissues from lung cancer, colorectal cancer, hepatocarcinoma, or renal cancer patients without COVID-19, as well as splenic tissues from trauma patients, were used as controls. The tissues were sectioned, fixed in formalin, and embedded in paraffin. COVID-19 or control sections were probed using a panel of 27 antibodies that enabled the detection of different lymphocyte types, cytokines, lymphocyte activation, and vascular and spatial structures of tissue cells (Supplementary Table 1). These sections were then subjected to imaging mass cytometry (IMC; Figure 1A). IMC is a progressive imaging technique that rather than analyzing single cells in suspension as in case of flow cytometry enables the detection of approximately 37 epitopes in paraffin-embedded tissue sections based on simultaneous antibody probing, thereby revealing the entire structure of the scanned tissue. Compared to immunohistochemistry or immunofluorescence, this technique can effectively reduce the background signal and crossover between fluorescence by employing isotopically applied metal tags and mass spectrometry. Meanwhile, the number of samples tested is reduced to one paraffin section, which greatly reduces sample consumption. IMC can not only improve the quality of experiments and results but also can facilitate subsequent bioinformatic analysis on the original specimen. Further, semi-quantitative data result in more accurate and upgraded information compared to those obtained with the simple qualitative analysis of images. Therefore, for accurate detection of the parameters, the adjustment of image parameters and the design and redacting of analysis algorithms are crucial. We used a t-SNE map to display 55,391 subsampled single cells from each PhenoGraph cluster identified in the heatmap image colored according to sample (control, patient 1, patient 2, patient 3), organ (lungs, intestine, spleen, liver, and kidneys), and cluster (Figures 1B–D). The z-scored mean marker expression of the panel markers was determined for each PhenoGraph cluster using heatmap analysis. Clusters and markers were grouped according to expression profiles (Figure 1E). Furthermore, we compared the percent relative abundances of cell clusters between the three patients and the control using a histogram (Figure 1F).

**Figure 1:**
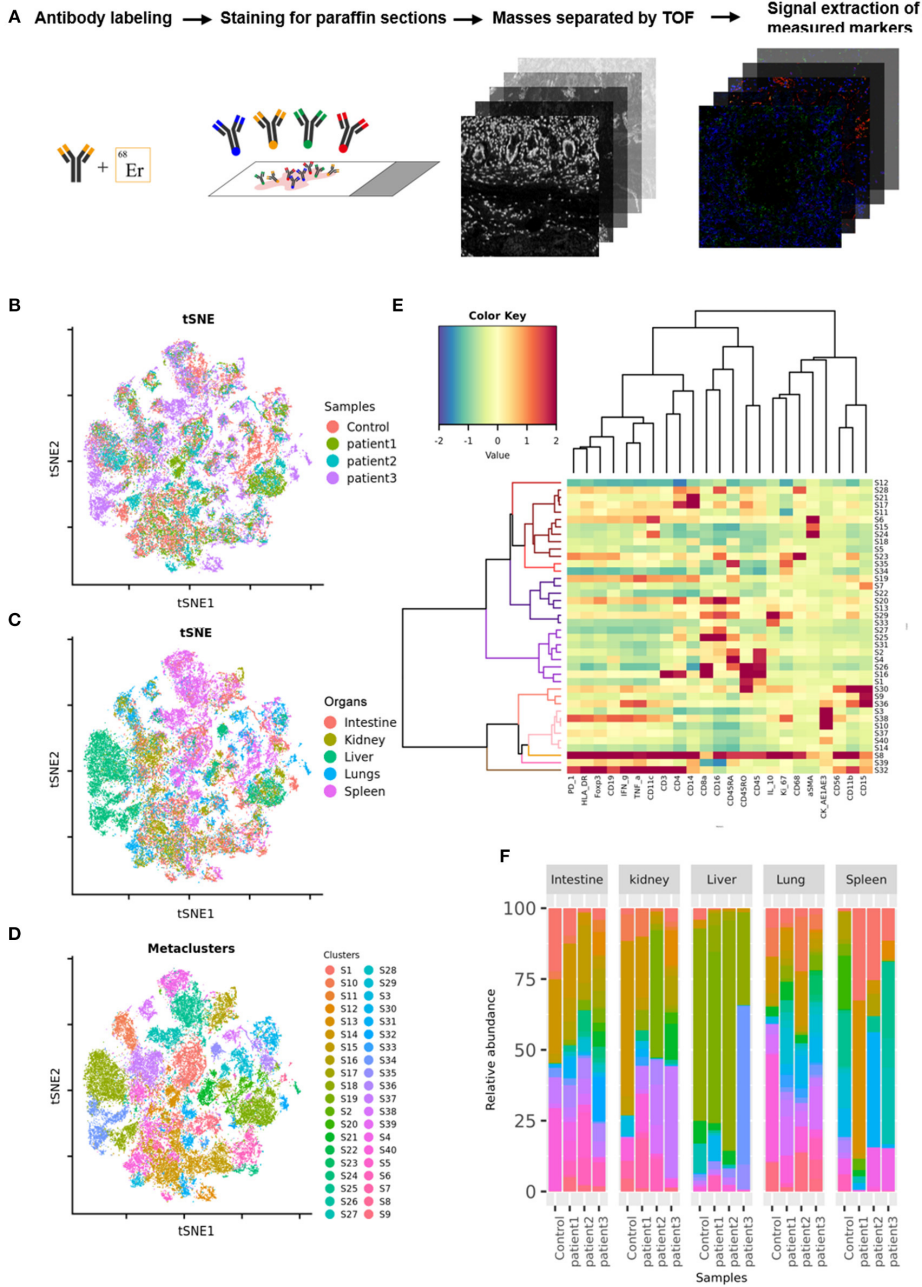
Imaging mass cytometry (IMC) of tissues from patients with coronavirus disease-2019 (COVID-19). **(A)** Schematic of IMC acquisition for multiplexed images from three patients with COVID-19 and controls. **(B)** t-SNE map displaying 55,391 subsampled single cells from each PhenoGraph cluster identified in heatmap images colored according to sample. **(C)** t-SNE map displaying 55,391 subsampled single cells from each PhenoGraph cluster identified in heatmap images colored according to organ. **(D)** t-SNE map displaying 55,391 subsampled single cells from each PhenoGraph cluster identified in heatmap images colored according to cluster. **(E)** Heatmap showing the z-scored mean marker expression of the panel markers for each PhenoGraph cluster. Clusters and markers are grouped according to expression profiles. **(F)** Bar plots showing the relative percent abundances of the cell clusters.

First, we found that monocytes (CD14 and CD68) infiltrated the lungs (Figures 2A,B) and intestines (Figures 3A,B) of patients with COVID-19. Moreover, CD11b^+^ macrophage, CD11c^+^ dendritic cell (DC), CD3^+^ CD56^+^ natural killer T (NKT) cell, and CD19^+^ B cell counts were higher in the lungs (Figures 2A,B) and intestines (Figures 3A,B) of patients with COVID-19 than in those of controls. These data suggest that the lung and intestine are the major targeted organs in COVID-19 and that SARS-CoV-2 infection induces a strong immune response in these organs. The difference among patient 1, patient 2 and patient 3 on the whole morphology of the lung and intestine tissues could be due to the different severity of acute lung and intestine injuries of these patients. However, changes in these immune cells differed in other organs. For example, in the liver of patients with COVID-19, no significant differences were observed in macrophage (CD14 and CD68) counts compared with those in controls (Supplementary Figures 1A,B), and counts of CD11b^+^ macrophages, CD11c^+^ DCs, and CD3^+^ CD56^+^ NKT cells were also higher than those in the control, the exception being patient 1 (Supplementary Figures 1A,B). In contrast, in the kidney, CD11b^+^ macrophage, CD11c^+^ DC, and CD19^+^ B cell counts were higher in patients 1 and 2 than those in the control, and patients 2 and 3 had more monocytes (CD14 and CD68) than the control (Supplementary Figures 2A,B). The counts of all immune cell were reduced in the spleens of patients with COVID-19 (Supplementary Figures 3A,B). In previous studies, flow cytometric analyses of peripheral blood mononuclear cells from symptomatic patients with COVID-19 revealed the significant influx of CD14^+^ monocytes (Xu et al., 2020; Zhang Y. et al., 2020), consistent with our findings. Furthermore, compared to those in control samples, the numbers of CD11b^+^ macrophages and CD11c^+^ DCs were elevated in the lungs, intestine, liver, and kidneys of all three patients with COVID-19 (Supplementary Figures 1, 2). However, CD11b^+^ macrophages and CD11c^+^ DCs were reduced in the spleens of patients with COVID-19 (Supplementary Figure 3), suggesting that the responses of CD11b^+^ macrophages and CD11c^+^ DCs to SARS-CoV-2 might be transferred from the spleen to the infected site or other damaged sites. Together, these results indicated that macrophages and DCs infiltrate the lungs—the major infection site of SARS-CoV-2—and other organs, including the intestine, kidneys, and liver, and this phenomenon is probably associated with multiorgan injuries. Moreover, NKT cells infiltrated the liver, lungs, and intestine of patients with COVID-19, and B cells infiltrated the kidneys, lungs, and intestines of these patients. These data suggest that unequal immune cell infiltration could induce tissue-specific immune responses; thus, monotherapy might not be sufficient for the treatment of patients with severe COVID-19.

**Figure 2:**
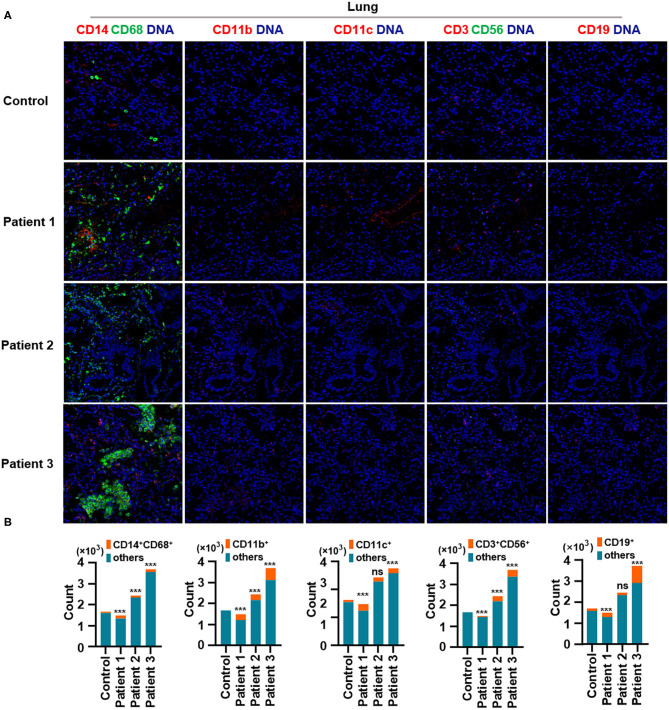
Immune cell responses to severe acute respiratory syndrome coronavirus 2 (SARS-CoV-2) in the lungs of patients with coronavirus disease-2019 (COVID-19). Representative imaging mass cytometry (IMC) for each panel, with different colors to distinguish panels; iridium-DNA staining is shown in blue. **(A)** Monocytes (CD14 and CD68), CD11b^+^ macrophages, CD11c^+^ dendritic cells (DCs), natural killer T (NKT) cells (CD3 and CD56), and B cells (CD19) in the lungs of controls and three patients with COVID-19. **(B)** Bar plot for the comparison of monocyte (CD14 and CD68), CD11b^+^ macrophage, CD11c^+^ DC, NKT cell (CD3 and CD56), and B cell (CD19) counts in the lungs of controls and three patients with COVID-19. The significance of differences between groups is shown in horizontal brackets and was assessed using Fisher's exact test. ***P < 0.01; ns, *P* > 0.01.

**Figure 3:**
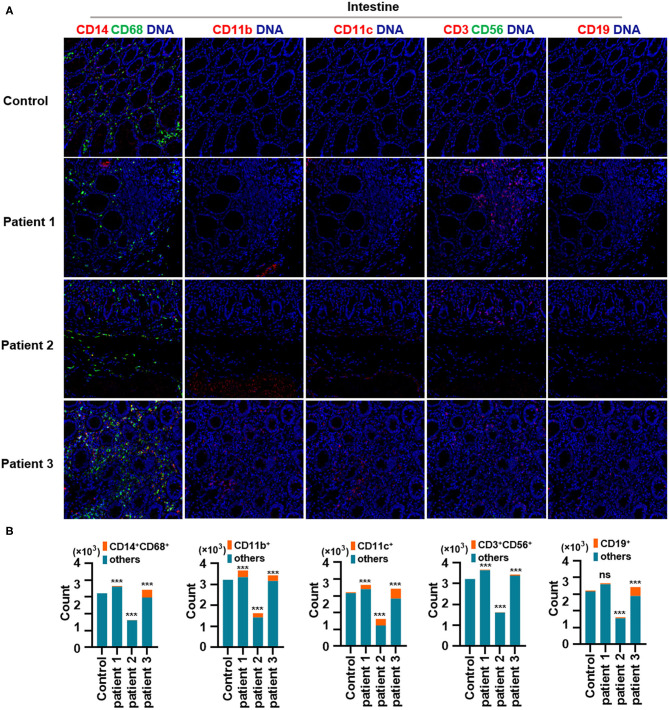
Immune cell responses to severe acute respiratory syndrome coronavirus 2 (SARS-CoV-2) in the intestine of patients with coronavirus disease-2019 (COVID-19). **(A)** Monocytes (CD14 and CD68), CD11b^+^ macrophages, CD11c^+^ dendritic cells (DCs), natural killer T (NKT) cells (CD3 and CD56), and B cells (CD19) in the intestine of controls and three patients with COVID-19. **(B)** Bar plot for the comparison of monocytes (CD14 and CD68), CD11b^+^ macrophages, CD11c^+^ DCs, NKT cells (CD3 and CD56), and B cells (CD19) in the intestine of controls and three patients with COVID-19. The significance of differences between groups is shown as horizontal brackets and was assessed using Fisher's exact test. ***P < 0.01; ns, *P* > 0.01.

Second, IL-10, an anti-inflammatory cytokine, plays a critical role in infection by limiting the immune response against pathogens, thereby preventing damage to the host. IL-10 is a well-known immunosuppressive cytokine (Mannino et al., 2015) that downregulates the expression of major histocompatibility complex class II (de Waal Malefyt et al., 1991). In our study, IL-10 production was elevated in the lungs of patients with COVID-19 and was found to be expressed by CD11b^+^ macrophages but not by CD11c^+^ DCs. Moreover, CD11b^+^ macrophages and CD11c^+^ DCs in the intestine showed higher IL-10 production (Figures 4A–C). In contrast, the expression of human leukocyte antigen DR isotype (HLA-DR) increased in the lungs and intestines of patients with COVID-19 but was found not to be expressed in CD11b^+^ macrophages or CD11c^+^ DCs (Figures 4D–F). Macrophages and DCs are the major antigen-presenting cells (APCs), and HLA-DR is the connecting link between the innate and adaptive immune responses. Our findings support the results of a previous study showing that IL-6 induces low HLA-DR expression and lymphopenia in patients with severe COVID-19 associated with immune dysregulation (Giamarellos-Bourboulis et al., 2020) and that the counts of circulating HLA-DR-positive cells were increased during convalescence in a patient with moderately severe COVID-19 (Thevarajan et al., 2020). Thus, severe respiratory failure (SRF)-aggravated pneumonia caused by SARS-CoV-2 might involve the following: a unique immunosuppressive pathway aimed at IL-10 overproduction via the inhibition of HLA-DR activation on APCs; high IL-10 secretion by macrophages and DCs.

**Figure 4:**
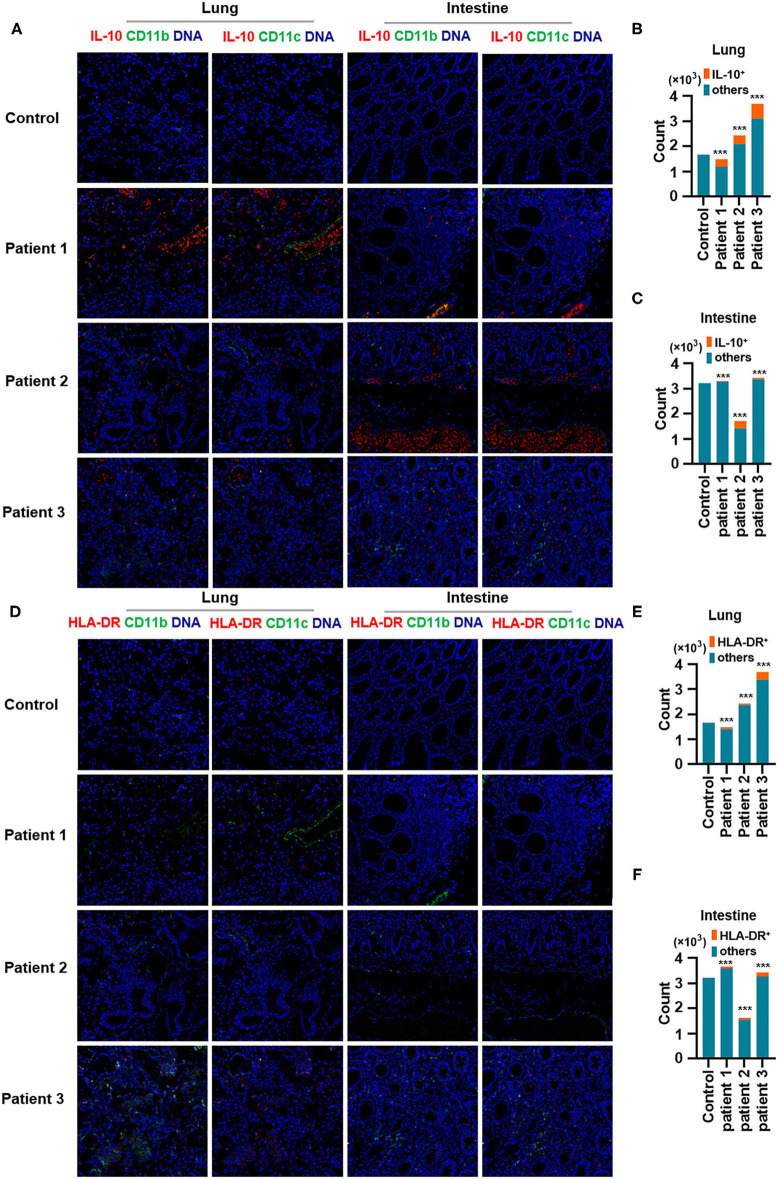
Production of IL-10 and expression of human leukocyte antigen DR isotype (HLA-DR) in the lungs and intestines of patients with coronavirus disease-2019 (COVID-19). **(A)** Imaging mass cytometry (IMC) for IL-10, CD11b, and CD11c in the lungs and intestine of controls and patients with COVID-19. Iridium-DNA staining is shown in blue, IL-10 staining is shown in red, and CD11b and CD11c staining is shown in green. **(B,C)** Bar plot for the comparison of IL-10, CD11b, and CD11c in the lungs and intestine of controls and three patients with COVID-19. **(D)** IMC for HLA-DR, CD11b, and CD11c in the lungs and intestine of controls and patients with COVID-19. Iridium-DNA staining is shown in blue, HLA-DR staining is shown in red, and CD11b and CD11c staining is shown in green. **(E,F)** Bar plot for the comparison of HLA-DR in the lungs and intestine of controls and patients with COVID-19. The significance of differences between groups is shown as horizontal brackets and was assessed using Fisher's exact test. ***P < 0.01; ns, *P* > 0.01.

Finally, we demonstrated the overproduction of tumor necrosis factor (TNF)-α in the lungs of all patients with COVID-19. In addition, TNF-α production in the intestines was consistent with changes in TNF-α expression in the lungs. Notably, TNF-α production was much higher in the liver of patient 1 and in the kidneys of all patients with COVID-19, as well as in the spleen of all patients with COVID-19 (Figures 5A,B). Previous studies have confirmed the role of TNF-α in promoting the pathogenesis of SARS-CoV infection through its receptors and suggested that the inhibition of TNF-α signaling might be leveraged as a treatment strategy (McDermott et al., 2016). However, extensive studies are needed to confirm the correlations between TNF-α and other inflammatory cytokines with respect to pathological inflammation. Circulating concentrations of IL-6 are significantly higher among patients infected with SARS-CoV-2, and IL-6 is a key pro-inflammatory factor leading to the generation of cytokine storms (Fu et al., 2020). Our study showed that TNF-α is a potent cytokine related to pathological inflammation in tissues—similar to IL-6—in patients with COVID-19.

**Figure 5:**
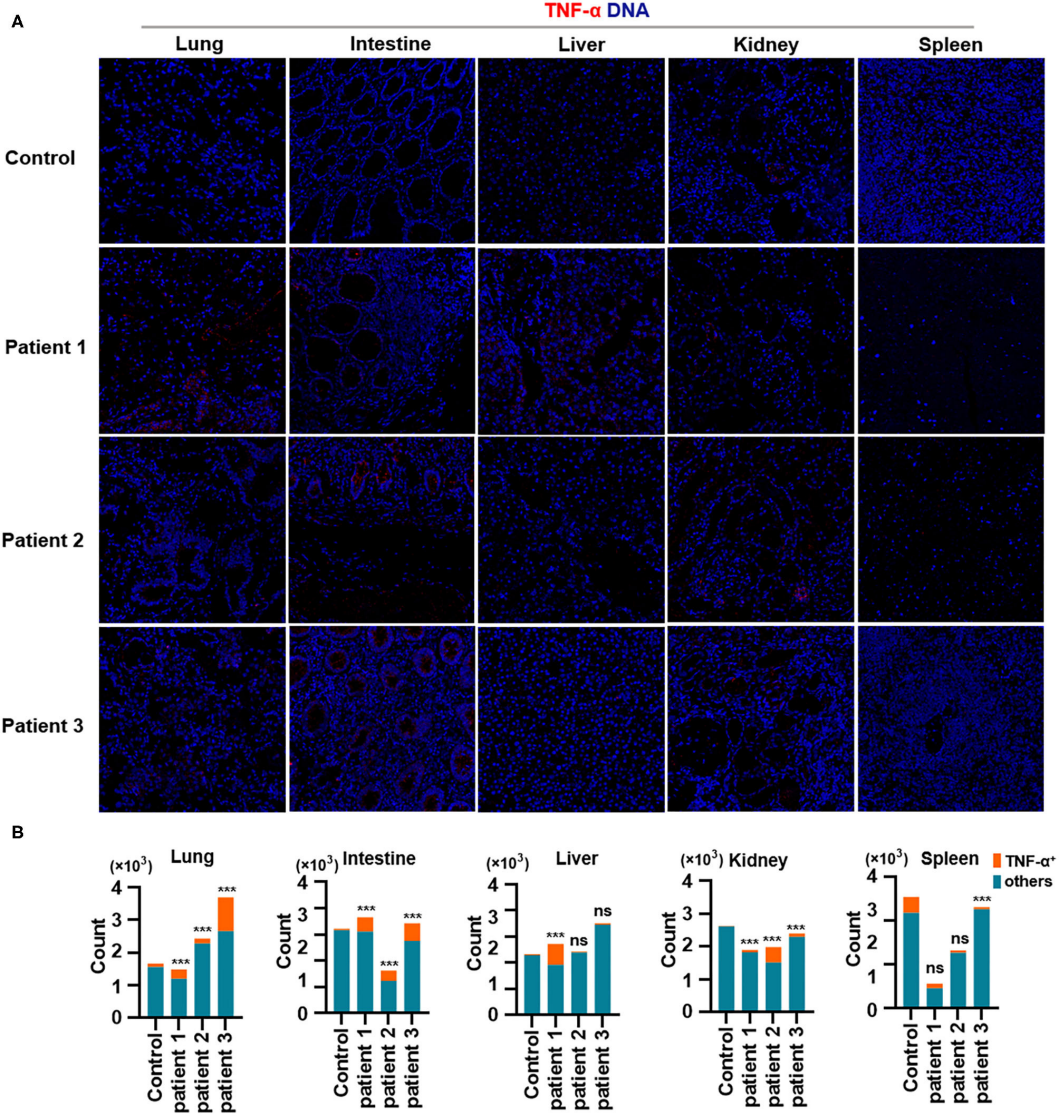
Production of TNF-α in tissues from patients with coronavirus disease-2019 (COVID-19). **(A)** Imaging mass cytometry (IMC) for TNF-α in the lungs, intestine, liver, kidneys, and spleen from controls or patients with COVID-19; iridium-DNA staining is shown in blue, and TNF-α staining is shown in red. **(B)** Bar plot for the comparison of TNF-α in the lungs and intestine of controls and three patients with COVID-19. The significance of differences between groups is shown as horizontal brackets and was assessed using Fisher's exact test. ***P < 0.01; ns, *P* > 0.01.

In summary, we showed for the first time that CD11b^+^ macrophages and CD11c^+^ DCs infiltrate the lungs and intestines, a phenomenon that is accompanied by overproduction of the immunosuppressive cytokine IL-10. However, the expression of HLA-DR on CD11b^+^ macrophages and CD11c^+^ DCs was low in the lungs and intestines of patients with COVID-19. Furthermore, we identified a unique signature of pathogenesis in patients with COVID-19, characterized by high levels of IL-10 production in the lungs and intestine, which might inhibit HLA-DR expression on macrophages or DCs, and TNF-α overproduction in the lungs, intestines, and other damaged tissues. This clear understanding of the delicate balance between antiviral and inflammatory innate immune programs will be essential to develop effective biomarkers and therapeutics for COVID-19.

## Experimental Section

### Tissues

Lung, intestine, spleen, liver, and kidney tissues were obtained by autopsy from patients with COVID-19 at Wuhan Jinyintan Hospital, Wuhan, Hubei in China. Patient 1 was a 53-year old woman, patient 2 was a 53-year-old woman, and patient 3 was a 60-year-old woman. In addition, control tissues were obtained from five different individuals; all samples were collected by surgical operation as follows: control 1 (75-year-old woman with no chronic basic disease) donated splenic tissue; control 2 (68-year-old man with hypertension) donated lung cancer tissue; control 3 (64-year-old woman with hypertension) donated kidney tissue; control 4 (72-year-old man with no chronic basic disease) donated liver tissue; control 5 (57-year-old woman with hypertension) donated intestine tissue (Supplementary Table 2). Tissues were oriented during staining to investigate their structures to the greatest extent.

All samples were treated in accordance with the biocontainment procedures required to process SARS-CoV-2-positive samples. All work performed in this study was approved by the Wuhan Jinyintan Hospital Ethics Committee (No. KY-2020-15.01). SARS-CoV-2 infection was diagnosed based on the New Coronavirus Pneumonia Prevention and Control Program (6th edition) published by the National Health Commission of China.

### Antibodies and Panel

The antibody panel included those related to lymphocyte types, cytokine expression, lymphocyte activation, and vascular and spatial structures of cells from other tissues. Descriptions of the antibodies, isotope tags, clones, and concentrations used for staining are provided in Supplementary Table 2. Metal-conjugated primary antibodies were applied using a Maxpar labeling kit (Fluidigm, USA). Concentration was determined using a Biotek instrument (Berten Instruments) and was adjusted to 0.5 μg/μL in Antibody Stabilization Solution (Candor Bioscience GmbH, Wangen, Germany). Antibodies were stored at 4°C for long-term storage.

### Tissue Antibody Labeling

Tissue samples were fixed in formalin and embedded in paraffin at Wuhan Jinyintan Hospital. Supplementary Table 1 shows the staining used for all samples. Slides were baked at 60°C in a slide dryer for 2 h, deparaffinized in xylene, and hydrated in decreasing concentrations of ethanol (100, 95, 80, 70%) for 5 min each. Next, 40 mL Antigen Retrieval Reagent-Basic (R&D Systems; diluted from 10× to 1×) was added to 50 mL conical tubes, and the tubes were incubated on a heating block (97°C) with loose lids. The tubes were then cooled to 60°C by monitoring the temperature for ~20 min. Next, the sections were blocked with 3% bovine serum albumin (BSA) for 45 min at room temperature (25°C) in a hydration chamber. The antibody cocktail was then prepared in 0.5% BSA in phosphate-buffered saline (PBS) PH 7.4 basic. Samples were probed overnight with antibodies at 4°C and then washed twice with 0.1% Triton X-100 prepared in PBS for 8 min with slow agitation in Coplin jars. Tissues were then stained with Intercalator-Ir (Fluidigm; cat. no. 201192A) in PBS for 30 min at room temperature to detect DNA and air dried before IMC measurements for at least 20 min (Figure 1A).

### IMC

For H&E staining, we selected the appropriate 500 × 500 μm location for scanning. IMC Images were acquired using a Hyperion Imaging System (Fluidigm). The largest square area was laser-ablated in a rastered pattern at 200 Hz, and preprocessing of the raw data was completed using commercial acquisition software (Fluidigm). IMC acquisition stability was monitored by the interspersed acquisition of an isotope-containing polymer (Fludigm). All successful image acquisitions were processed using MCDViewer, CellProfilor, and HistoCAT.

### Single-Cell Identification

The original MCD file was converted to TIFF format using imctools (https://github.com/BodenmillerGroup/imctools). Data were segregated into single cells using Cellprofiler v3.1.8 (Carpenter et al., 2006). Briefly, Fluidigm DNA markers were designed to identify nuclei, and CD3, CD4, CD8, CD45, pan-cytokeratin, and α-smooth muscle actin were used to identify membranes. The data were combined and analyzed using Cellprofier to identify single-cell object masks. The mask (TIFF file) containing the cell location and boundary was obtained. Even with high-quality segmentation, imaging of tissue segments yielded single-cell data from tissue slices and overlapping cell fragments that did not always capture the nucleus of a cell; therefore, nucleus-mismatched signals could be assigned to neighboring cells in densely packed areas. This could lead to rare cases in which the data assigned to one cell contained marker expression from the neighboring cells.

### Data Transformation and Normalization

Single-cell marker expression was measured using histoCat v1.75 (Schapiro et al., 2017), multiplied by a factor of 10^7^ to yield values larger than 1, and then log-transformed. The single-cell data were censored at the 95th percentile to remove the outliers, and *z*-scored cluster means were visualized using heat maps. For *t*-SNE and PhenoGraph, the data were normalized with Harmony (Korsunsky et al., 2019).

### Clustering

Single cells from the large cohort were clustered into groups according to their phenotypic similarity using PhenoGraph, unsupervised clustering, and an aggregation of these clusters into larger groups based on their markers. The data were clustered to detect and separate rare cell subpopulations. PhenoGraph (v.2.0) (Levine et al., 2015) was used with 15 of the nearest neighbors. The resulting 40 clusters were aggregated into larger groups following hierarchical clustering (Euclidean distance and Ward's linkage) of their mean marker correlations. Multiscale bootstrap resampling was used to assess the uncertainty of each subtree (R package pvclust, v.2.0).

### Barnes-Hut T-SNE

For visualization, high-dimensional single-cell data were reduced to two dimensions using the non-linear dimensionality reduction algorithm t-SNE (Kobak and Berens, 2019). We applied the Barnes-Hut implementation of *t*-SNE to Harmony-normalized data with default parameters (perplexity = 42).

### Neighborhood Analysis

To identify significantly enriched or depleted pairwise neighbor interactions between cell types, the Cellprofier MeasureObjectNeighbors module and neighborhood (https://github.com/BodenmillerGroup/neighboRhood) functions were used to perform a permutation-test-based analysis of the spatial single-cell neighborhood. Neighboring cells were defined as those within 4 pixels (4 μm). *P-*values smaller than 0.01 were considered significant.

## Data Availability Statement

The original contributions presented in the study are included in the article/supplementary materials, further inquiries can be directed to the corresponding author/s.

## Ethics Statement

All work performed in this study was approved by the Wuhan Jinyintan Hospital Ethics Committee (No. KY-2020-15.01). Diagnosis of SARS-CoV-2 infection was based on the New Coronavirus Pneumonia Prevention and Control Program (6th edition) published by the National Health Commission of China. The patients/participants provided their written informed consent to participate in this study. Written informed consent was obtained from the individual(s) for the publication of any potentially identifiable images or data included in this article.

## Author Contributions

CW, JX, SW, SP, JZ, YH, and MH performed experiments with the help of DW, QYan, XY, YYang, TS, XZo, RL, YL, RY, and YaW. YQ, YuW, D-YZ, QYao, YYan, XZh, and YS analyzed the data. CW, JX, SW, MH, XZh, and YS wrote the manuscript. QYao, YYan, XZh, and YS designed and supervised the overall study.

## Conflict of Interest

SW, YYang, YL, RY, and YYan were employed by company Gencode Diagnostics Inc., Beijing, China.

The remaining authors declare that the research was conducted in the absence of any commercial or financial relationships that could be construed as a potential conflict of interest.

## Supplementary Material

Supplementary Figure 1 Immune cell responses to severe acute respiratory syndrome coronavirus 2 (SARS-CoV-2) in the liver of patients with coronavirus disease-2019 (COVID-19). Representative imaging mass cytometry (IMC) for each panel, with different colors to distinguish panels; iridium-DNA staining is shown in blue. **(A)** Monocytes (CD14 and CD68), CD11b^+^ macrophages, CD11c^+^ dendritic cells (DCs), natural killer T (NKT) cells (CD3 and CD56), and B cells (CD19) in the liver of controls and three patients with COVID-19. **(B)** Bar plot for the comparison of monocytes (CD14 and CD68), CD11b^+^ macrophages, CD11c^+^ DCs, NKT cells (CD3 and CD56), and B cells (CD19) in the liver of controls and patients with COVID-19. The significance of differences between groups is shown as horizontal brackets and was assessed using Fisher's exact test. ^***^*P* < 0.01; ns, *P* > 0.01.

Supplementary Figure 2 Immune cell responses to severe acute respiratory syndrome coronavirus 2 (SARS-CoV-2) in the kidneys of patients with coronavirus disease-2019 (COVID-19). Representative imaging mass cytometry (IMC) for each panel, with different colors to distinguish panels; iridium-DNA staining is shown in blue. **(A)** Monocytes (CD14 and CD68), CD11b^+^ macrophages, CD11c^+^ dendritic cells (DCs), natural killer T (NKT) cells (CD3 and CD56), and B cells (CD19) in the kidneys of controls and patients with COVID-19. **(B)** Bar plot for the comparison of monocytes (CD14 and CD68), CD11b^+^ macrophages, CD11c^+^ DCs, NKT cells (CD3 and CD56), and B cells (CD19) in the kidneys of controls and patients with COVID-19. The significance of differences between groups is shown as horizontal brackets and was assessed using Fisher's exact test. ^***^*P* < 0.01; ns, *P* > 0.01.

Supplementary Figure 3 Immune cell responses to severe acute respiratory syndrome coronavirus 2 (SARS-CoV-2) in the spleen of patients with coronavirus disease-2019 (COVID-19). Representative imaging mass cytometry (IMC) for each panel, with different colors to distinguish panels; iridium-DNA staining is shown in blue. **(A)** Monocytes (CD14 and CD68), CD11b^+^ macrophages, CD11c^+^ dendritic cells (DCs), natural killer T (NKT) cells (CD3 and CD56), and B cells (CD19) in the spleen of controls and patients with COVID-19. **(B)** Bar plot for the comparison of monocyte (CD14 and CD68), CD11b^+^ macrophage, CD11c^+^ DC, NKT cell (CD3 and CD56), and B cell (CD19) counts in the spleen of controls and patients with COVID-19. The significance of differences between groups is shown as horizontal brackets and was assessed using Fisher's exact test. ^***^*P* < 0.01; ns, *P* > 0.01.

Supplementary Table 1 Descriptions of antibodies, isotope tags, clones, and concentrations used for staining.

Supplementary Table 2 Descriptions of patients with COVID-19 and controls.
