# Supplementary figures and images for: Imaging Mass Cytometric Analysis of Postmortem Tissues Reveals Dysregulated Immune Cell and Cytokine Responses in Multiple Organs of COVID-19 Patients
Source: Front Microbiol. 2020 Dec 23;11:600989. doi: 10.3389/fmicb.2020.600989 (PMC7785801; doi:10.3389/fmicb.2020.600989)

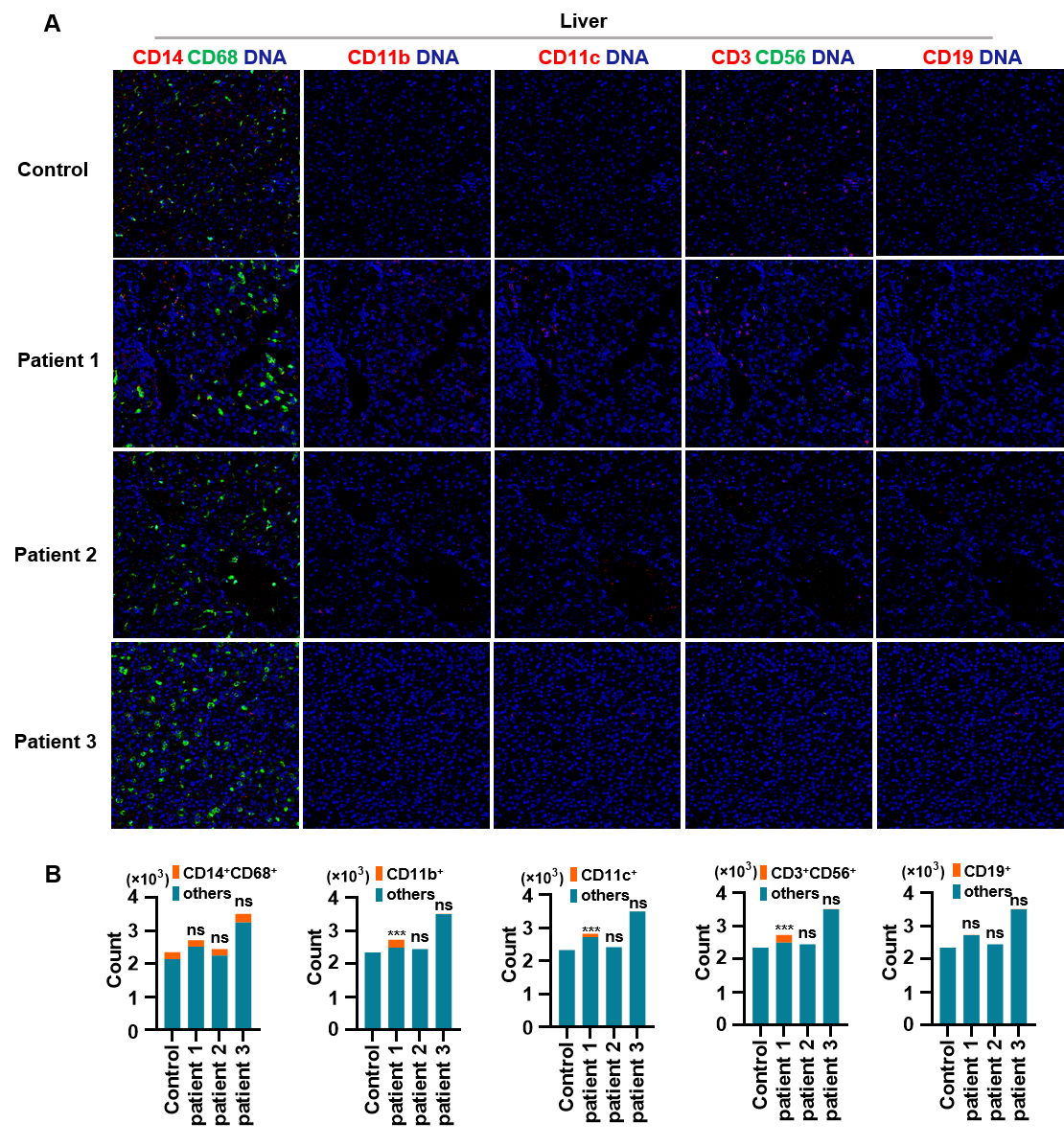

Supplement: Supplementary Figure 1 — Immune cell responses to severe acute respiratory syndrome coronavirus 2 (SARS-CoV-2) in the liver of patients with coronavirus disease-2019 (COVID-19). Representative imaging mass cytometry (IMC) for each panel, with different colors to distinguish panels; iridium-DNA staining is shown in blue. (A) Monocytes (CD14 and CD68), CD11b+ macrophages, CD11c+ dendritic cells (DCs), natural killer T (NKT) cells (CD3 and CD56), and B cells (CD19) in the liver of controls and three patients with COVID-19. (B) Bar plot for the comparison of monocytes (CD14 and CD68), CD11b+ macrophages, CD11c+ DCs, NKT cells (CD3 and CD56), and B cells (CD19) in the liver of controls and patients with COVID-19. The significance of differences between groups is shown as horizontal brackets and was assessed using Fisher's exact test. ***P < 0.01; ns, P > 0.01. [file Image_1.TIF]

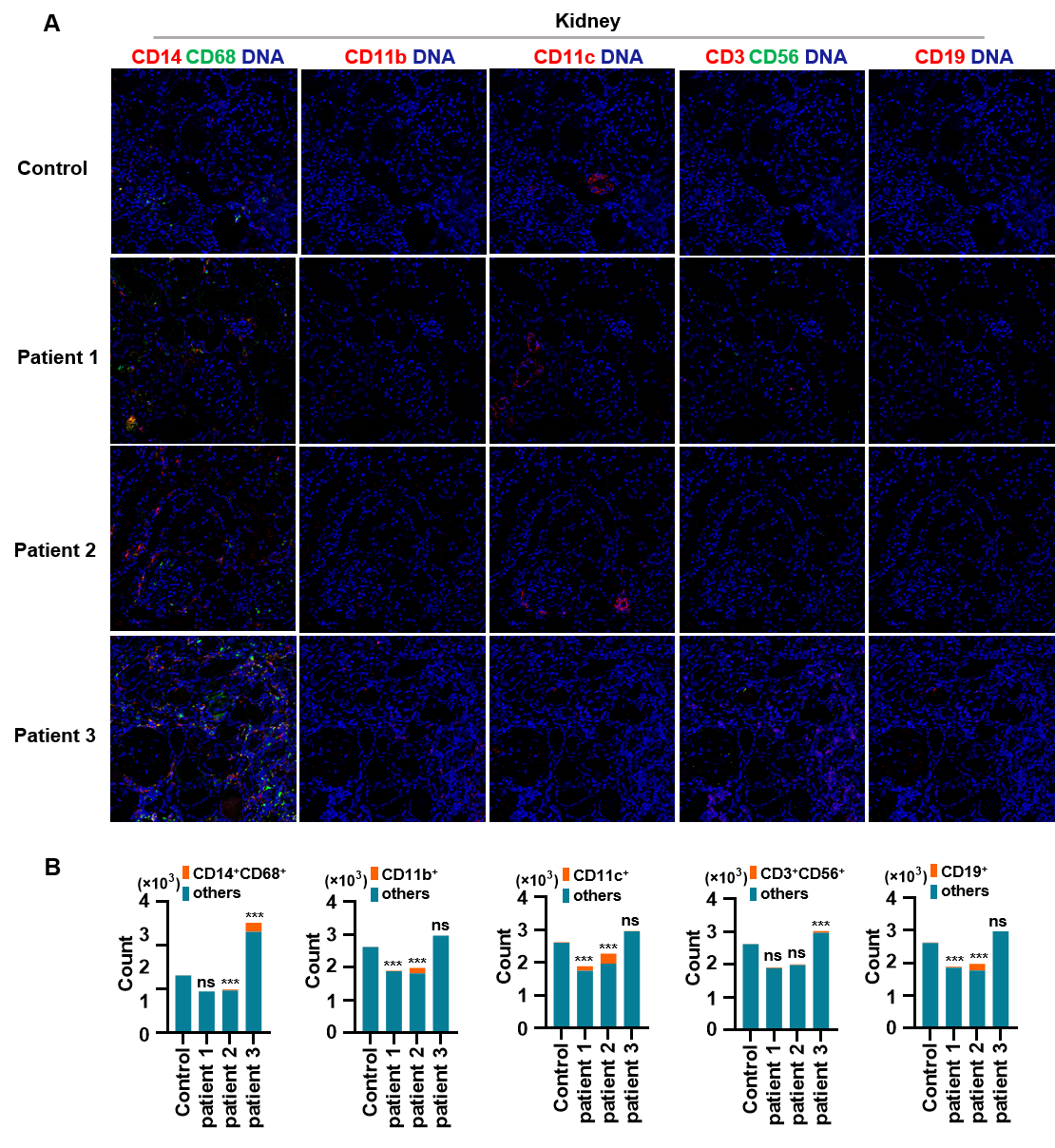

Supplement: Supplementary Figure 2 — Immune cell responses to severe acute respiratory syndrome coronavirus 2 (SARS-CoV-2) in the kidneys of patients with coronavirus disease-2019 (COVID-19). Representative imaging mass cytometry (IMC) for each panel, with different colors to distinguish panels; iridium-DNA staining is shown in blue. (A) Monocytes (CD14 and CD68), CD11b+ macrophages, CD11c+ dendritic cells (DCs), natural killer T (NKT) cells (CD3 and CD56), and B cells (CD19) in the kidneys of controls and patients with COVID-19. (B) Bar plot for the comparison of monocytes (CD14 and CD68), CD11b+ macrophages, CD11c+ DCs, NKT cells (CD3 and CD56), and B cells (CD19) in the kidneys of controls and patients with COVID-19. The significance of differences between groups is shown as horizontal brackets and was assessed using Fisher's exact test. ***P < 0.01; ns, P > 0.01. [file Image_2.TIF]

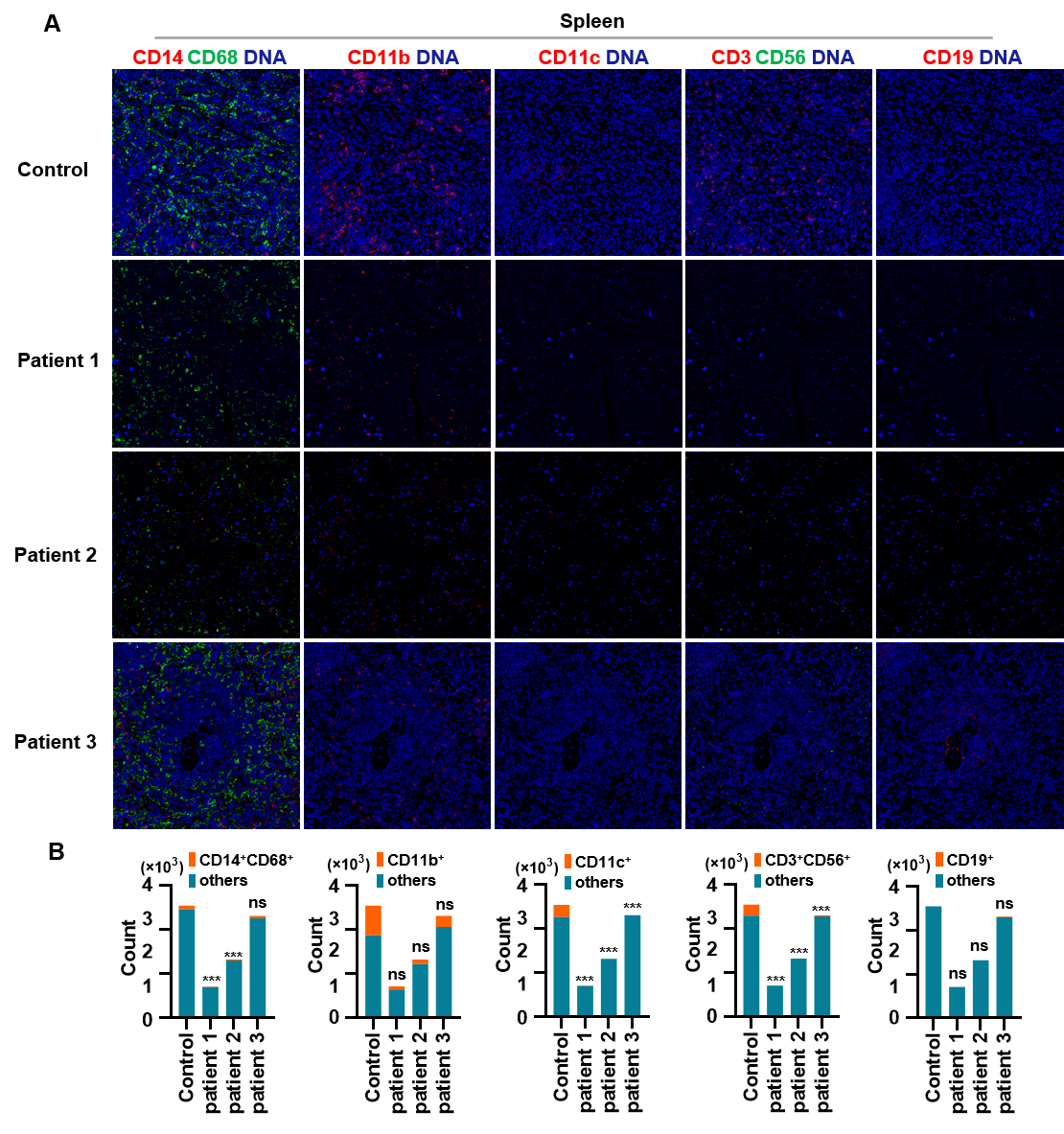

Supplement: Supplementary Figure 3 — Immune cell responses to severe acute respiratory syndrome coronavirus 2 (SARS-CoV-2) in the spleen of patients with coronavirus disease-2019 (COVID-19). Representative imaging mass cytometry (IMC) for each panel, with different colors to distinguish panels; iridium-DNA staining is shown in blue. (A) Monocytes (CD14 and CD68), CD11b+ macrophages, CD11c+ dendritic cells (DCs), natural killer T (NKT) cells (CD3 and CD56), and B cells (CD19) in the spleen of controls and patients with COVID-19. (B) Bar plot for the comparison of monocyte (CD14 and CD68), CD11b+ macrophage, CD11c+ DC, NKT cell (CD3 and CD56), and B cell (CD19) counts in the spleen of controls and patients with COVID-19. The significance of differences between groups is shown as horizontal brackets and was assessed using Fisher's exact test. ***P < 0.01; ns, P > 0.01. [file Image_3.TIF]
